# Data of the subatomic resolution structure of glucose isomerase complexed with xylitol inhibitor

**DOI:** 10.1016/j.dib.2023.109916

**Published:** 2023-12-06

**Authors:** Yongbin Xu, Ki Hyun Nam

**Affiliations:** a Department of Bioengineering, College of Life Science, Dalian Minzu University, Dalian 116600, China; b Key Laboratory of Biotechnology and Bioresources Utilization of Ministry of Education, College of Life Science, Dalian Minzu University, Dalian 116600, China; c College of General Education, Kookmin University, Seoul 02707, South Korea

**Keywords:** Glucose isomerase, X-ray crystallography, Subatomic resolution, Data collection, Radiation damage, Data processing

## Abstract

Glucose isomerase (GI) is a crucial enzyme in industrial processes, including the production of high-fructose corn syrup, biofuels, and other renewable chemicals. Understanding the mechanisms of GI inhibition by GI inhibitors can offer valuable insights into enhancing production efficiency. We previously reported the subatomic resolution structure of *Streptomyces rubiginosus* GI (SruGI) complexed with a xylitol inhibitor, determined at 0.99 Å resolution, was reported. Structural analysis showed that the xylitol inhibitor is partially bound to the M1 binding site at the SruGI active site, enabling it to distinguish the xylitol-bound and -free state of SruGI. This structural information demonstrates that xylitol binding to the M1 site causes a conformational change in the metal binding site and the substrate binding channel of SruGI. Herein, detailed information on data collection and processing procedures of the subatomic resolution structure of the SruGI complexed with xylitol was reported.

Specifications Table

**Table:** 

Subject	Biological sciences
Specific subject area	Structural Biology
Data format	Raw
Type of data	X-ray diffraction data, Table, Image, Graph, Figure
Data collection	Synchrotron: Pohang Light Source II (PLS-II) Beamline: 11C Detector: Pilatus 6 M (DECTRIS). Data collection temperature: 100 K Data processing program: Xia2
Data source location	Institution: Kookmin University City/Town/Region: Seoul Country: Republic of Korea
Data accessibility	1. Raw data diffraction images Repository name: ZENODO Data identification number: https://doi.org/10.5281/zenodo.7948815 Direct URL to data: https://zenodo.org/record/7948815 2. Structure factor and coordinate Repository name: Protein Data Bank Data identification number: https://doi.org/10.2210/pdb8WDG/pdb Direct URL to data: https://www.rcsb.org/structure/8WDG
Related research article	Y. Xu, K.H. Nam, Xylitol binding to the M1 site of glucose isomerase induces a conformational change in the substrate binding channel, Biochem. Biophys. Res. Commun. (2023) [1]https://doi.org/10.1016/j.bbrc.2023.09.087

## 1 Value of the Data

• Diffraction data for the subatomic resolution of GI complexed with the xylitol inhibitor were obtained.
• Diffraction images were analyzed to reduce radiation-damaged information.
• The subatomic resolution structure of GI complexed with xylitol was determined at 0.99 Å resolution.
• Two partial conformational changes in the metal binding site at the active site and the substrate binding channel were observed using subatomic resolution data.

## 2 Data Description

The subatomic crystal structure of SruGI complexed with the xylitol inhibitor was determined at a resolution of 0.99 Å using a microfocusing synchrotron X-ray beam [1]. This high-precision subatomic resolution structural information provides detailed insight into the molecular mechanism underlying the conformational changes in the metal binding site at the active site and the substrate binding channel of SruGI.

**Fig. 1:**
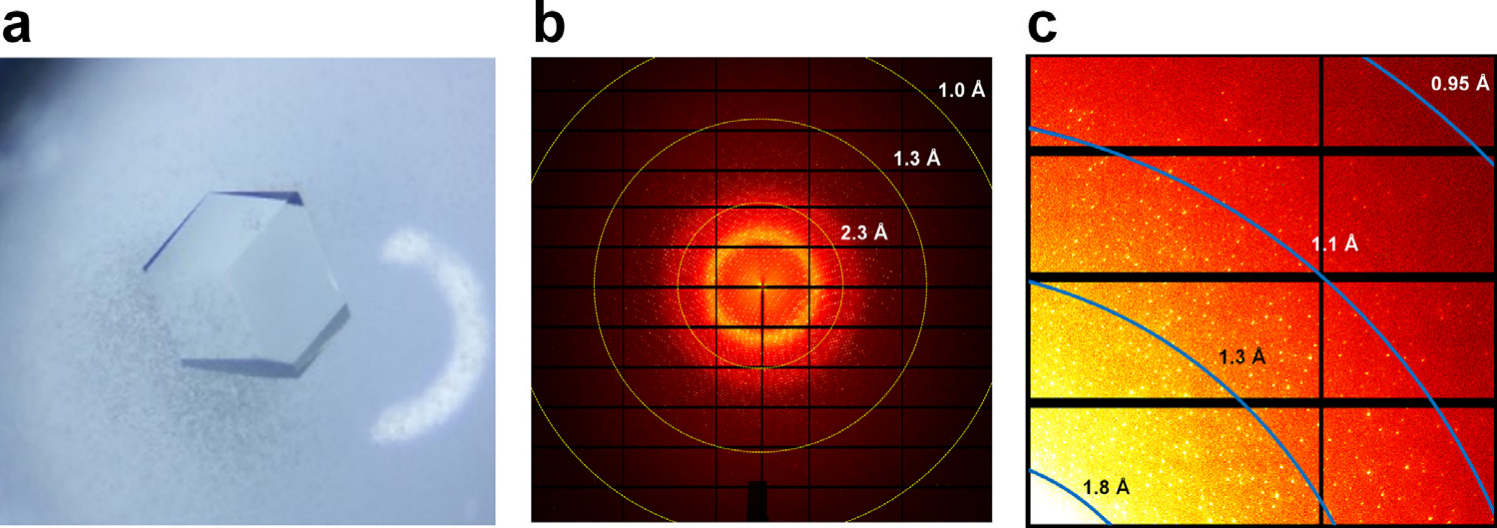
Crystallographic results of the subatomic resolution structure of SruGI-xylitol. (a) Photograph of the SruGI crystal. (b) Subatomic diffraction pattern of SruGI. (c) Close-up view of the high-resolution area of SruGI.

SruGI, purchased commercially and containing SruGI crystals, had varying sizes, ranging from a few to hundreds of micrometers. Although large SruGI crystals are suitable for collecting high-resolution diffraction data, their appearance is a non-uniform crystal surface morphology, potentially causing increased mosaicity and inaccurate structural information. Therefore, to obtain high-quality crystals, a re-crystallization experiment was conducted using a SruGI solution. Subsequently, high-quality SruGI crystals measuring 500 × 500 × 700 µm³ were obtained and used to collect diffraction data (Fig. 1a). To obtain the structure using the xylitol inhibitor, SruGI crystals were immersed in a xylitol solution containing the crystallization solution and xylitol for approximately 30 s. Subsequently, after a 5-s soak in the cryoprotectant solution, the crystals were mounted on a goniometer under a 100 K environment at a beamline of 11C from a Pohang Light Source II. Diffraction data were obtained by rotating the crystal and exposing it to synchrotron X-rays. In total, 360 diffraction images were obtained, spanning 360°. The diffraction limit of the crystal was < 1 Å (Fig. 1b). The raw X-ray diffraction images have been deposited in the ZENODO repository (http://zenodo.org). There are 360 image files, named SruGI-15keV3_0001 to SruGI-15keV3_0360, in the CBF file format. The numbers 1–360 in the images denote the order of the data obtained during exposure to synchrotron X-rays. The header of the CBF file contains information on the detector and beamline parameters used for data collection, including wavelength during data collection, sample-to-detector distance, detector pixel size, and beam center.

**Fig. 2:**
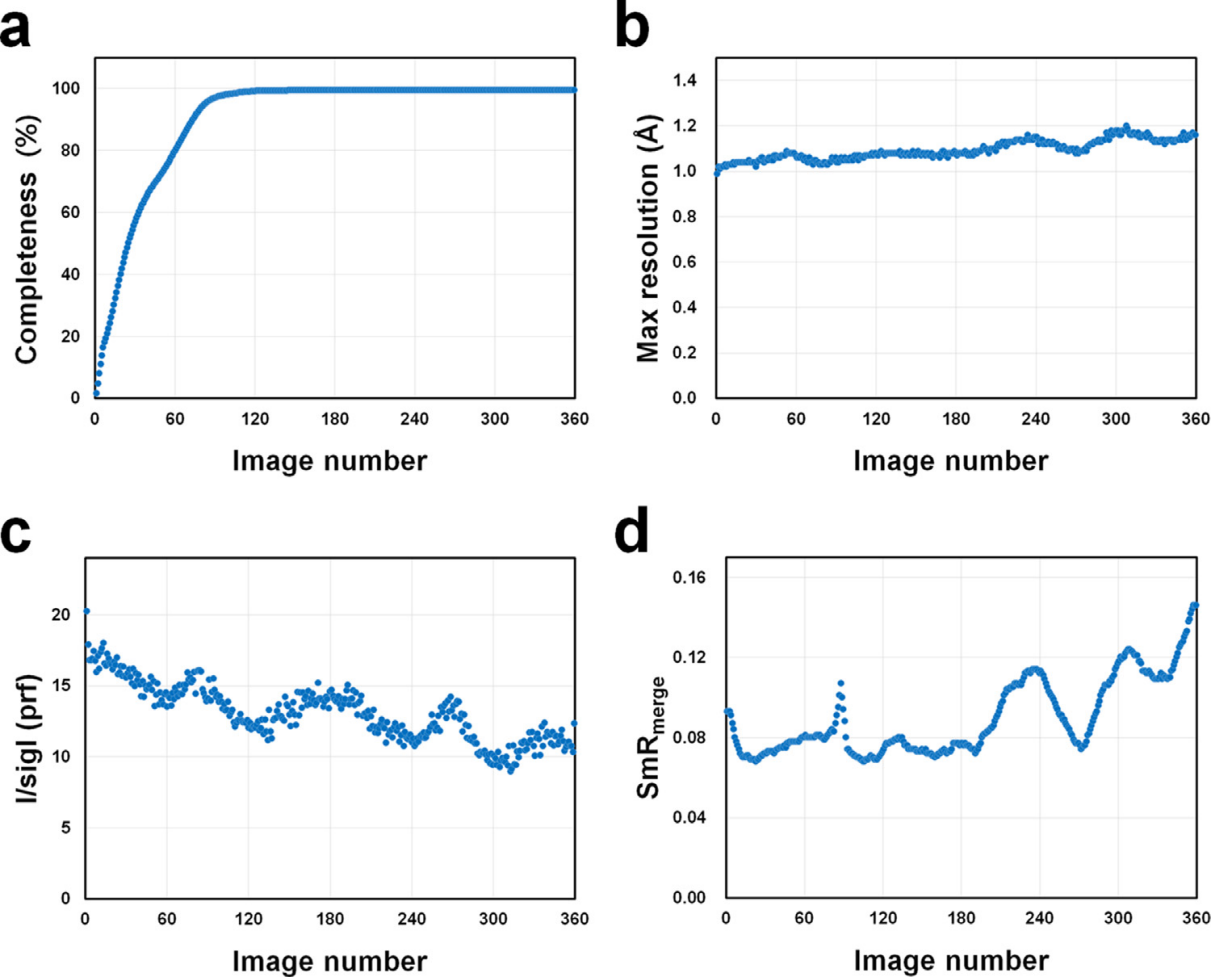
Data processing statistics for SruGI of (a) completeness, (b) maximum resolution, (c) I/sigI values with profile fitting, and (d) SmR_merge_.

At the outset, 360 diffraction images were processed up to a resolution of 0.99 Å (Table 1). Although these data processing statistics were reasonable for determining the subatomic resolution structure, concerns were raised regarding possible X-ray radiation damage to the SruGI crystal. During data collection, as the crystal was rotated on the goniometer, its center was continuously exposed to the X-ray beam. Furthermore, because the X-ray beam penetrated the SruGI crystal, theoretically, the crystal volume along the path would be re-exposed to X-rays after a 180° rotation [2]. Consequently, the crystal diffraction data might contain information on radiation damage, potentially devalidating the structural information accuracy [2], [3], [4], [5]. To mitigate the effect of radiation damage in SruGI complexed with xylitol, crystallographic statistics for each diffraction image were analyzed (Fig. 2). Data completeness reached 99.5% before the 180° rotation (Fig. 2a). Max-resolution refers to within 1 Å of the initial image and early 1 Å thereafter; however, the maximum resolution measured after 180° rotation exceeded 1.08 Å (Fig. 2b). The I/sigma values, considering the profile fitting patterns, indicated a consistent decreasing trend as exposure to X-rays increased (Fig. 2c). Specifically, I/sigI values at 181°–360° appear to be reduced by approximately 30%–40% compared with 1°–180°. Furthermore, when examining SmR_merge_, the overall values remained below 0.1 before the 180° rotation. However, a clear increasing trend was observed after a 180° rotation (Fig. 2d). This trend was consistent with a previous report on radiation damage in thaumatin using a microfocusing beamline [2]. Considering the data collection statistics and previous reports, the diffraction data between 1° and 180° images were used for the final subatomic resolution structure determination of the SruGI complexed with xylitol. Table 1 shows detailed data processing statistics.

**Table 1:** Detailed data collection statistics.

Data	SruGI complexed with Xylitol					
Processed images	1–180			1–360		
Space group	I 2 2 2			I 2 2 2		
a, b, c (Å)	92.61, 98.56, 102.08			92.68, 98.61, 102.13		
α, β, γ (°)	90.00, 90.00, 90.00			90.00, 90.00, 90.00		
Resolution range (Å)	Overall	Low	High	Overall	Low	High
High resolution limit	0.99	2.69	0.99	0.99	2.69	0.99
Low resolution limit	40.02	40.05	1.01	40.73	40.77	1.01
Total observations	1,554,628	86,555	45,393	3,116,024	173,502	89,706
Total unique	255,970	13,342	12,132	256,684	13,379	12,177
Completeness (%)	99.5	99.9	94.6	99.6	99.9	94.9
Multiplicity	6.1	6.5	3.7	12.1	13.0	7.4
I/sigma	112.2	43.4	1.0	12.8	40.7	1.1
R_merge_(I)	0.058	0.029	0.947	0.088	0.048	1.668
R_meas_(I)	0.064	0.031	1.104	0.091	0.050	1.794
R_pim_(I)	0.025	0.012	0.553	0.026	0.014	0.644
CC half	0.999	0.999	0.561	0.999	0.999	0.565
Wilson B factor (Å^2^)	7.272			7.616

**Fig. 3:**
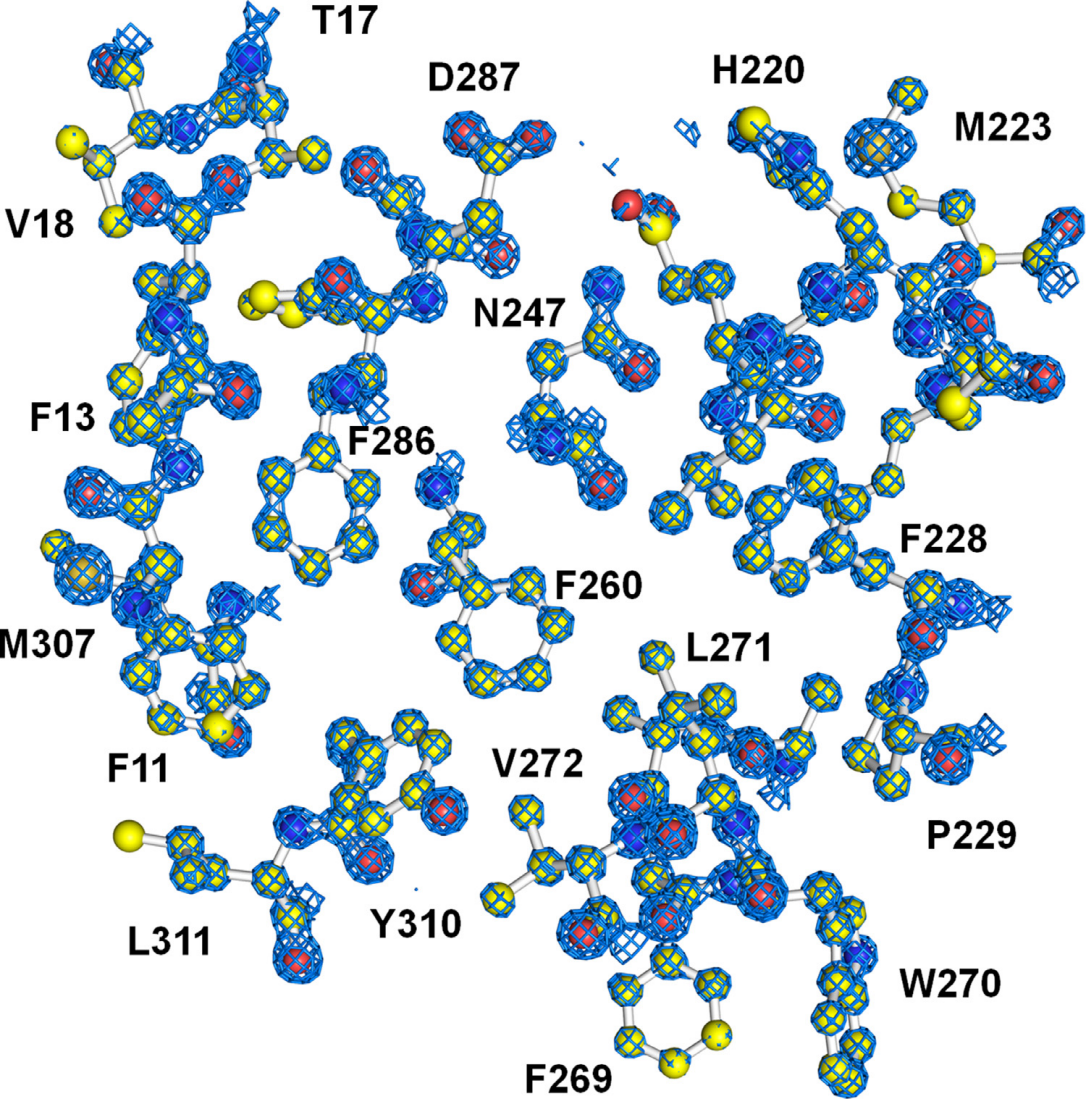
2Fo-Fc electron density map (blue mesh, 5σ) of the subatomic resolution structure of SruGI complexed with xylitol.

The phasing problem was solved using the molecular replacement method, and the model was constructed based on the 2Fo-Fc (3–4σ) and Fo-Fc (± 3 σ) electron density maps. The subatomic resolution data allowed for the accurate positioning of most atoms in the 2Fo-Fc electron density map, which was counted at 5 σ (Fig. 3), except for the metal binding and substrate entrance sites. The binding of xylitol to the M1 site of SruGI was verified by OMIT map calculation [1]. However, electron density map and temperature factor analysis revealed that xylitol binding to the M1 site of SruGI is not fully occupied, resulting in two distinct SruGI conformations, as xylitol-bound and xylitol-free states. In a previous study, when xylitol was bound to the M1 metal binding site, a conformational change occurred at the M1 and M2 metal sites, resulting in the release of the metal at the M2 site [6]. Similarly, in the subatomic resolution structure of SruGI-xylitol, partial positive and negative Fo-Fc electron density maps were observed around Asp255 and Glu186, among the amino acids interacting with the M2 site metal. This showed a similar trend in electron density map changes for the amino acids surrounding the M2 site owing to xylitol binding. Furthermore, it was observed in the subatomic resolution electron density map that the structural change of Glu186 affected the conformation of Pro25 and Phe26 amino acids from the neighboring protomers in the tetrameric SruGI. These confirmed that conformational changes occurred from Asp255, Glu181, Pro25, and Phe26 because of the partial binding of xylitol to the SruGI M1 site. The atomic-resolution data revealed structural changes in the metal and substrate binding sites in response to xylitol binding and its absence. It also elucidated the molecular mechanism of the structural changes in SruGI due to inhibitor binding. The final model, including two conformational changes, was constructed based on the electron density map. The structural factors and coordinates for this subatomic resolution structure of SruGI complexed with xylitol were deposited in the Protein Data Bank (PDB) under the code 8WDG.

## 3 Experimental Design, Materials and Methods

The sample preparation, crystallization, data collection, and processing procedures for SruGI have been described [1]. X-ray diffraction data were obtained using a microfocusing beamline 11C from a Pohang Light Source II (PLS-II) in the Republic of Korea [7]. To obtain the xylitol-bound state, SruGI crystals were soaked in an inhibitor solution containing Tris–HCl, pH 8.0, 15% (v/v) polyethylene glycol 400, 100 mM MgCl_2_, and 20 mM xylitol for 30 min and then immersed in inhibitor solution supplemented with 20% (v/v) glycerol for 5 s. Cryoprotected SruGI crystals soaked in xylitol solution were mounted on the goniometer under a 100 K liquid nitrogen stream. The X-rays were microfocused using a KB mirror, resulting in vertical and horizontal X-ray sizes of approximately 3.5 µm and 8.5 µm (full-width half maximum), respectively. During crystal diffraction screening, diffraction data from SruGI crystals with resolutions beyond 1 Å were observed. To obtain subatomic resolution data, an X-ray energy of 15 keV was used in this experiment (wavelength: 0.895 Å). Diffraction data were obtained using a Pilatus 6 M detector. Each image contains diffraction information for a 1° oscillation. Diffraction images, including the Bragg peaks, were indexed, integrated, and scaled using the Xia2 program [8]. Electron density maps were obtained through the molecular replacement (MR) method using the MOLREP program [9]. The coordinates of the xylitol-free state of SruGI (PDB code: 7CJP) [10] determined at a 2.5 Å resolution were used as the MR search model. The partial xylitol binding state was verified using an OMIT map, and manual model building was performed using COOT [9]. The final model structure was refined using REFMAC5 [11]. The raw diffraction images have been deposited at ZENODO (http://zenodo.org) under the accession https://doi.org/10.5281/zenodo.7948815. X-ray diffraction data (PDBx/mmCIF), structure coordinates (PDBx/mmCIF, PDBML, and PDB), and a validation report were deposited in the Protein Data Bank (http://rcsb.org) under accession code 8WDG.

## Limitations

Not applicable.

## Ethics Statement

This work meets the ethical requirements for publication in this journal. This work does not involve human subjects, animal experiments, or any data collected from social media.

## CRediT authorship contribution statement

**Yongbin Xu:** Formal analysis, Writing – review & editing. **Ki Hyun Nam:** Data curation, Formal analysis, Funding acquisition, Validation, Visualization, Writing – original draft, Funding acquisition.

## Data Availability

Structure factors and coordinates (Original data) (Protein Data Bank) Diffraction images (Original data) (ZENODO)
